# Engineered Mesoporous Silica-Based Nanoparticles: Characterization of Surface Properties

**DOI:** 10.3390/ma17133352

**Published:** 2024-07-06

**Authors:** Antonio Grisolia, Marzia De Santo, Manuela Curcio, Palmira Alessia Cavallaro, Catia Morelli, Antonella Leggio, Luigi Pasqua

**Affiliations:** 1 Department of Environmental Engineering, University of Calabria, Via P. Bucci, 87036 Arcavacata di Rende, CS, Italy; antonio.grisolia@unical.it; 2 Department of Pharmacy, Health and Nutritional Sciences, University of Calabria, 87036 Arcavacata di Rende, CS, Italy; marzia.desanto@unical.it (M.D.S.); manuela.curcio@unical.it (M.C.); catia.morelli@unical.it (C.M.); antonella.leggio@unical.it (A.L.)

**Keywords:** mesoporous silica nanoparticles, grafting, surface properties, zeta-potential, external functionalization

## Abstract

Mesoporous silica-based nanomaterials have emerged as multifunctional platforms with applications spanning catalysis, medicine, and nanotechnology. Since their synthesis in the early 1990s, these materials have attracted considerable interest due to their unique properties, including high surface area, tunable pore size, and customizable surface chemistry. This article explores the surface properties of a series of MSU-type mesoporous silica nanoparticles, elucidating the impact of different functionalization strategies on surface characteristics. Through an extensive characterization utilizing various techniques, such as FTIR, Z-potential, and nitrogen adsorption porosimetry, insights into the surface modifications of mesoporous silica nanoparticles are provided, contributing to a deeper understanding of their nanostructure and related interactions, and paving the way to possible unexpected actionability and potential applications.

## 1. Introduction

Since their synthesis in the early 1990s, mesoporous silica-based nanomaterials have piqued the interest of researchers across various fields, from catalysis to nanomedicine and nanotechnology applications [1]. Numerous materials have been developed since the introduction of mesoporous solids. Their biocompatibility, stability, and surface modification capabilities render them highly promising candidates for surmounting limitations in diverse research domains [2,3]. The starting architectures are porous solids with regular pore diameter and a high specific surface area, so they show the ability to extract molecules from body fluids [4], to take up and hold enzymes [5], and load a drug releasing them according to a strategy that exclusively targets cancer cells [6]. Through the engineering of mesoporous silica nanoparticles with customized surface functionalities, precise manipulation of their interactions with systems can be attained, facilitating specific interactions while mitigating, e.g., in the field of nanomedicine, potential side effects [1]. Thus, mesoporous silica nanoparticles, with their robust structure and adjustable surface chemistry, provide an ideal platform for the nanostructuring of matter at the nanoscale, thus allowing the development of nanostructured functional materials or nanodevices for different applications [7].

Mesoporous silica-based nanostructured materials can find extensive application in environmental remediation, significantly reducing the presence of harmful compounds in environmental matrices (water, air, and soil) [8,9,10]. Indeed, due to the wide array of harmful compounds stemming from various industrial activities such as pharmaceutical, metallurgical, and petroleum industries, applying traditional decontamination treatments (coagulation, flocculation, filtration, and others) proves challenging [11,12,13], while remediation using hybrid nanostructured systems based on mesoporous silica can allow highly specific interactions potentially able to address various criticalities [14].

Taking the hybrid nanomaterials under study as a reference, it can be observed, in the literature, that there are examples of similar functionalizations being used for the capture of environmental pollutants. For instance, Youssef et al. employed a facile synthesis protocol to surface functionalize folic acid onto mesoporous silica-based nanomaterials, thus creating hybrid systems useful for capturing Al^3+^ ions from aqueous matrices [15]. Almethen et al. utilized similar functionalizations for the capture of organic pollutants, such as methylene blue, an extensively prevalent and environmentally harmful dye [16]. Nanomaterials functionalized with APTES can serve as adsorbents for both organic and inorganic pollutants, as demonstrated by studies like those of Jadhav et al., who applied these hybrid nanomaterials to capture Cr (VI), and by Cueto-Diaz et al., who investigated their ability to adsorb CO_2_ [17,18,19].

Salman and colleagues, starting from the unique physicochemical characteristics of silica-based nanoparticles, assert that mesoporous silica modified with molecules containing -NH2, -COOH, and other functional groups exhibit promising and efficient metal adsorption capacity. They emphasize that the mechanisms of adsorption are diverse, involving ion-pair formation, solid-phase extraction, ligand exchange, electrostatic interactions, and surface complexations [20].

It has been demonstrated that nanoparticles functionalized with APTES exhibit a higher adsorption capacity, towards organic compounds such as fulvic acid, compared to non-functionalized nanoparticles [21].

Wang et al. further modified the aminated nanoparticles by covalently binding them with tryptophan (Trp). They demonstrated that these hybrid nanoparticles can adsorb ovalbumin (ova) from complex biological matrices with higher adsorption efficiency and capacity compared to the native aminated MSNs [22].

Regarding the functionalization methodology, Tripaldi et al. have exploited the reactivity of the external amino group of APTES surface-bound to mesoporous silica materials. They employed this reactivity for the reaction with succinic anhydride derivatives to create further functionalized hybrid materials [23].

The materials engineering approach can, according to versatile procedures, provide mesoporous silica-based nanodevices useful for both bionanotechnology and nanomedicine purposes [24,25,26,27]. The conventional approach often involves systemic administration of therapies, leading to indiscriminate distribution throughout the body and potential adverse effects on healthy tissues. To overcome these hurdles, nanotechnology has emerged as an innovative frontier, offering unprecedented opportunities for nanomedicine and, prospectively, multitargeting drugs in precision medicine solutions [28].

The aim and purpose of this research work is the description of the surface properties of mesoporous silica-based nanomaterials subjected to different post-synthesis protocols and how these properties vary. We have followed the variation of surface properties of different samples after single and multi-step modification procedures of the silica surface, as well as surfactant extraction protocols used during the synthesis of the MSN_AS_ precursor nanomaterials.

According to our current knowledge, there are no existing papers, in open literature, that investigate the variation of surface properties of mesoporous silica-based nanoparticles following successive grafting operations, including multi-step processes, and surfactant removal, while assessing their zeta potential and porosimetric characteristics. The evaluation of these properties holds paramount importance for potential applications both in drug delivery and environmental contexts. The accessibility of pores and material surfaces, as well as potential electrostatic interactions to which the hybrid material may be subjected, will determine the behavior of the nanomaterial, thus establishing its suitability for the envisioned application. We believe that this study can improve the awareness in the relationship between causes (modifications) and effects (properties variations), which is necessary background for a material chemist, when a nanostructured material is developed according to the bottom-up approach.

Amino-propyl triethoxysilane (APTES) and folic acid (FA) were introduced onto the nanoparticles and the samples (MSN-AP and FOL-MSN) were characterized either before and after the template extraction in water (FOL-MSN-EXT); FOL-MSN-EXT was functionalized with 3-glycidoxypropyltrimethoxysilane to obtain the corresponding diol (FOL-MSN-DIOL) and again with APTES (FOL-MSN-NH_2_) and characterized.

FOL-MSN-NH_2_ was functionalized with succinic acid to obtain FOL-MSN-COOH, and finally, FOL-MSN-COOH was functionalized with hydrazine monohydrate to form FOL-MSN-HYD and characterized.

The starting material (MSN_AS_) was extensively characterized using various techniques, including SEM, TEM, XRD, nitrogen adsorption-desorption porosimetry, infrared spectroscopy, and Z-potential analysis. The surface properties of the functionalized nanomaterials were explored using Z-potential and FT-IR infrared analyses. Finally, the pore structure of the several different synthesized samples derived from the starting material were characterized by nitrogen adsorption porosimetry, while their surface was investigated via FTIR spectroscopy and zeta-potential analysis to assess how these properties varied depending on the reactivities of the introduced groups.

Therefore, leveraging the characteristics of mesoporous silica-based nanomaterials, along with the surface functionalization potential of their external surface and mesopores, we can produce hybrid materials suitable for both environmental applications, such as the adsorption of organic or inorganic pollutants, and nanomedicine applications, including imaging or drug delivery.

## 2. Materials and Methods

### 2.1. Chemicals and Reagents

The reagents utilized in this study were obtained commercially and were of analytical grade. They were used as received without any further purification. Solvents underwent purification procedures in accordance with standard laboratory protocols and were freshly distilled prior to usage. Specifically, Triton X-100, a neutral polyoxyethylene octylphenyl ether, tetraethylorthosilicate (TEOS), (3-aminopropyl)-triethoxysilane (APTES), folic acid (FOL), and diisopropylcarbodiimide (DIC), were procured from MERCK/Sigma-Aldrich (Milan, Italy). Additionally, ethanol, diethyl ether, 1,4-dioxane, dimethylformamide (DMF), tetrahydrofuran (THF), trifluoroacetic acid (TFA), and acetic acid were sourced from VWR. Dimethyl sulfoxide (DMSO) and cyclohexane were acquired from Merck, while triethylamine was purchased from Carlo Erba (Milan, Italy). Ultrapure water was obtained using the MilliQs water system from Millipore (Burlington, MA, USA).

**Scheme 1:**
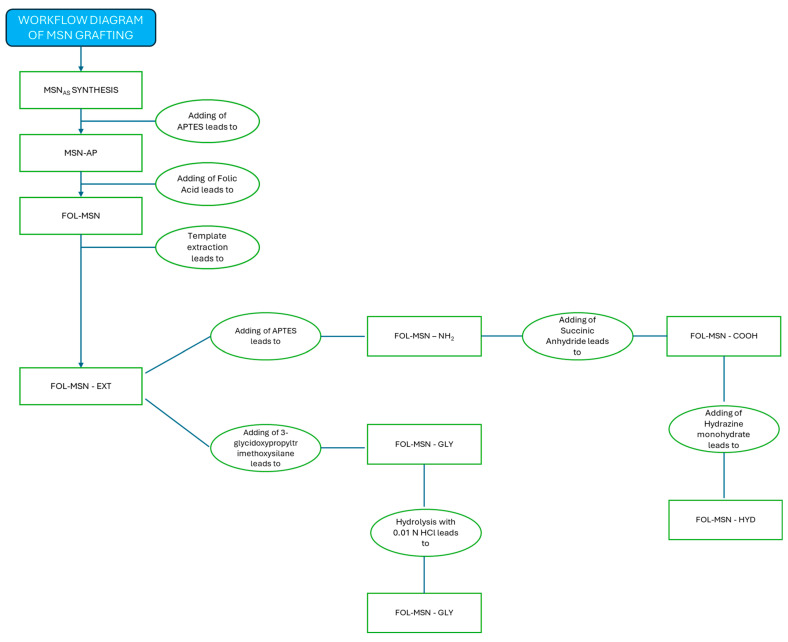
Workflow diagram of MSN grafting protocols.

Scheme 1 summarizes the workflow of MSN grafting protocols described in more detail below.

#### MSN_AS_ Synthesis

The synthesis procedure commenced by dissolving 21 g of Triton X-100 surfactant in 230 g of ultrapure water at 25 °C, a process taking approximately 4 h. Concurrently, a solution containing 22 g of tetraethyl orthosilicate (TEOS) dissolved in 9.8 g of cyclohexane was prepared. Introducing the TEOS solution into the surfactant gradually established a biphasic interface, maintaining a molar composition of TEOS:Cyclohexane:Triton X-100:H_2_O = 1:1.08:0.32:120.

This composite solution was subjected to slow stirring for 30 days at room temperature to facilitate nanoparticle precipitation in the polar phase (H_2_O). Following the separation of the non-polar component, the precipitate underwent filtration and rinsing with ultrapure water. Subsequently, the sample was dried in an oven at 70 °C for 24 h, resulting in the formation of a white powder.

### 2.2. Grafting Protocol

#### 2.2.1. Synthesis of Amine-Functionalized MSNs

A solution containing 28.02 g (0.126 moles) of APTES in 33.04 mL of ethanol was added to a suspension of 8 g of MSNs in 27.88 mL of ethanol. The synthesis was left under stirring at room temperature for 2 days. The resulting suspension was filtered and washed once with ethanol and twice with ultrapure water. The MSN-AP sample was then placed in an oven at 70 °C for 24 h.

#### 2.2.2. Synthesis of FOL-MSN

Folic acid (1.11 g, 2.51 mmol) was completely dissolved in DMSO (19.30 mL); after that, triethylamine (0.554 mL, 3.9 mmol), DIC (1.11 mL, 7 mmol), and MSN-AP (5.50 g) were added. The obtained suspension was stirred at room temperature for 40 h. Finally, the mixture was filtered and washed with dimethylformamide, dioxane, diethyl ether, and ultrapure water (once for each solvent). The resultant yellow powder (6.98 g) was dried and stored in sealed containers protected from light.

#### 2.2.3. Template Extraction Protocol

The surfactant within the pores was removed using 1 g of material in 0.33 L of ultrapure water at room temperature. The number of extractions to perform to reach a complete surfactant removal was established by monitoring (TGA) the total mass loss of small amounts of samples subjected to additional extraction steps, until a constant value was reached. Then, the solution of the last extraction was filtered, and the obtained FOL-MSN sample was washed with 1,4-dioxane and dried at 45 °C overnight.

#### 2.2.4. Synthesis of FOL-MSN-DIOL

The inner pores of FOL-MSN were functionalized with 3-glycidoxypropyltrimethoxysilane. A suspension of FOL-MSN (1 g) in 1,4-dioxane (30 mL) 3-glycidoxypropyltrimethoxysilane (2 mL) was added. The reaction mixture was kept under stirring at room temperature for 18 h. Then, the mixture was washed with dioxane and THF and filtered through nylon filters before the resulting powder was dried at 318.15 K. The recovered product (FOL-MSN-GLY) was subsequently treated with a 0.001 N HCl solution (pH 2–3). The mixture was stirred at room temperature for 10 h. After this time, the reaction mixture was washed with ultrapure water and THF, filtered, and dried at 318.15 K to afford FOL-MSN-DIOL.

#### 2.2.5. Synthesis of FOL-MSN-NH_2_

FOL-MSN-EXT was dissolved in 1,4-dioxan (0.05 g/mL) and then APTES (2.2 g/g MSNs) was added. After 18 h under stirring, the suspension was filtered and the solid washed with 1,4-dioxan and THF. The nanoparticles (FOL-MSN-NH_2_) were dried at 318.15 K overnight.

#### 2.2.6. Synthesis of FOL-MSN-COOH

Succinic anhydride (0.005 g/mL) was solubilized in dry dioxane, then FOL-MSN-NH_2_ was added. The mixture was stirred at room temperature for 24 h; next, filtration and washing with dry dioxan and dry dichloromethane occurred. The synthesized sample, FOL-MSN-COOH, was dried at 318.15 K overnight.

#### 2.2.7. Synthesis of FOL-MSN-HYD

The recovered FOL-MSN-COOH (2.19 × 10^−4^ molCOOH) powder was suspended in DMSO (0.02 g/mL); then, the EDC (2 molCOOH) and sulfo-NHS (2∙molCOOH) were added and the mixture was stirred for 1 h. After that, hydrazine monohydrate (21.3 mL, 4.38 × 10^−4^) was added to the reaction mixture that was left stirring at room temperature for 24 h. The final FOL-MSN-HYD sample was recovered after filtration and washing with DMSO, dioxane and dichloromethane.

### 2.3. Instrumental Characterizations

**TGA–DSC**

TG-DSC analyses were carried out using a Netzsch STA 449 instrument (Netzsch, Selb, Germany) in the temperature range of 293.15 K to 1123.15 K, with a heating rate of 10 K min^−1^ under ambient air conditions with a flow rate of 10 mL min^−1^, sample pan Al_2_O_3_–85 µL open, and DSC range 0–5000 µV.

**Zeta Potential**

Zeta-potential values were determined using the Zeta-sizer ZS (Malvern Instruments Ltd., Malvern, UK) at 298.15 ± 0.1 K. Measurements were conducted in ultrapure water, with a viscosity of 0.8872 cp and a refractive index of 1.330 at 298.15 K. The thermostatting time was 120 s, the dielectric constant of the dispersing medium was 78.5, and 3 replicate measurements were taken for each sample. Approximately 10 mg of each sample was dispersed in 10 mL of pre-filtered MilliQ water using 0.2 μm filters with a pH = 7.1 ± 0.1. The dispersion was vortexed for 1 min and then sonicated in an ultrasonic bath (45 kHz, 80 W) for 10 min to completely disaggregate any present aggregates. The negative potential was measured at a temperature of 298.15 ± 0.1 K in ultrapure water with a viscosity of 0.8872 cp and a refractive index of 1.330 at 298 K. The thermostatting time was 120 s, the dielectric constant of the dispersing medium was 78.5, and 3 replicate measurements were taken for each sample. To ensure better dispersion, the solution was vortexed for 1 min and then sonicated in an ultrasonic bath (45 kHz, 80 W) for 10 min to completely disaggregate any present aggregates.

**TEM—Transmission Electron Microscopy**

Transmission electron microscopy images were obtained with a Jeol 1400 Plus electron microscope (JEOL Ltd., Tokyo, Japan), operating at an acceleration voltage of 80 kV.

**FT-IR**

Fourier-transform infrared (FT-IR) spectra were obtained with an Infrared Spectrometer (FT/IR-4600 FT-8IR, Jasco Corporation; Tokyo, Japan).

**N_2_—Adsorption Porosimetry**

The specific surface area and the average pore width of all samples were evaluated according to the Brunauer-Emmett-Teller (BET) method, by physical adsorption measurements of nitrogen, using a Micromeritics Tristar II plus (Micromeritics Instruments Corporation, Norcross, GA, USA) with the pre-treatment degassing system Micromeritics FlowPrep 060 (Micromeritics, Norcross, GA, USA). Samples were pretreated at 120 °C in the degassing system for 150 min before analysis.

**X-ray Diffraction**

XRD measurements were performed with a MiniFlex Rigaku (Rigaku Holding Corporation, Tokyo, Japan), scan interval 0.3 < 2theta < 10, with a scan speed of 0.005 °/s with a Cu Kα (λ = 1.54059 Å) radiation.

## 3. Results

The MSN mesoporous silica was synthesized using a biphasic emulsion method employing a neutral polyethyleneoxide-type surfactant at room temperature [29]. After filtration, the synthesized material, (MSN_AS_), underwent characterization using X-ray powder diffraction, TEM, SEM, N_2_-adsorption porosimetry, thermogravimetry, FTIR spectroscopy, and Z-potential measurements.

### 3.1. MSN_AS_ Characterization

**Figure 1:**
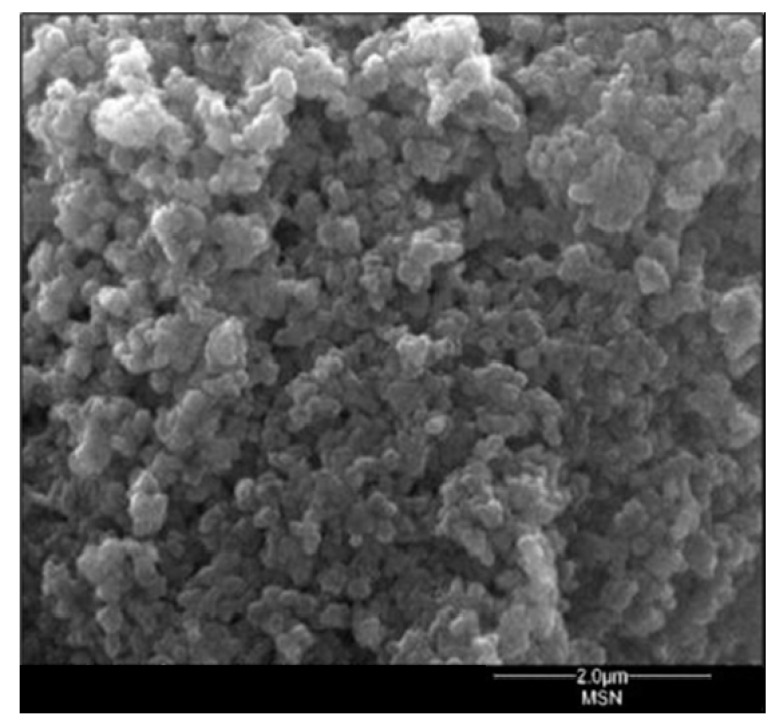
SEM micrograph image of MSN_AS_.

**Figure 2:**
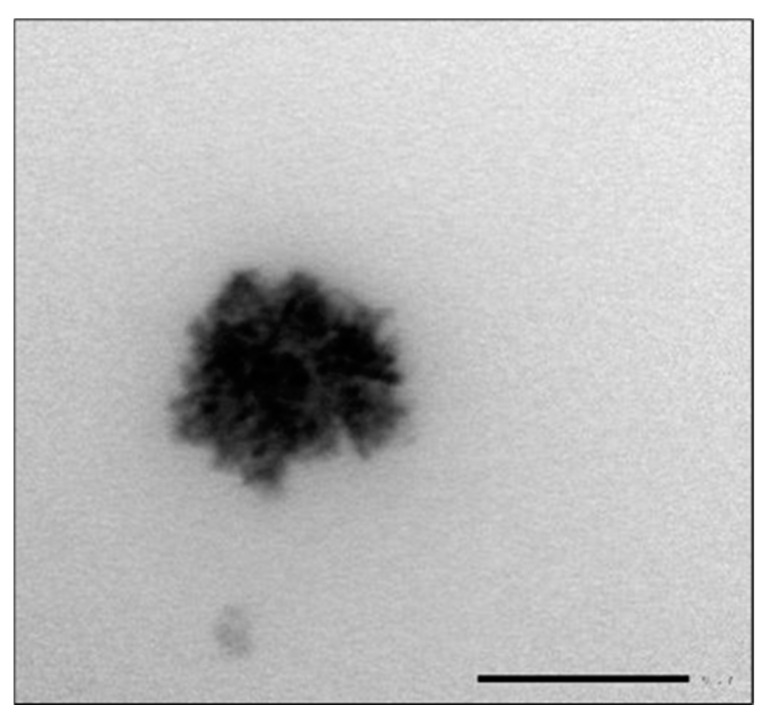
TEM micrograph image of MSN_AS_ (scale bar 100 nm).

The MSNs synthesized (MSN_AS_) were characterized by both transmission and scanning electron microscopy (TEM, SEM). They had an irregular and spheroidal shape. Their dimensions ranged from 80 to 120 nm (Figure 1 and Figure 2).

**Figure 3:**
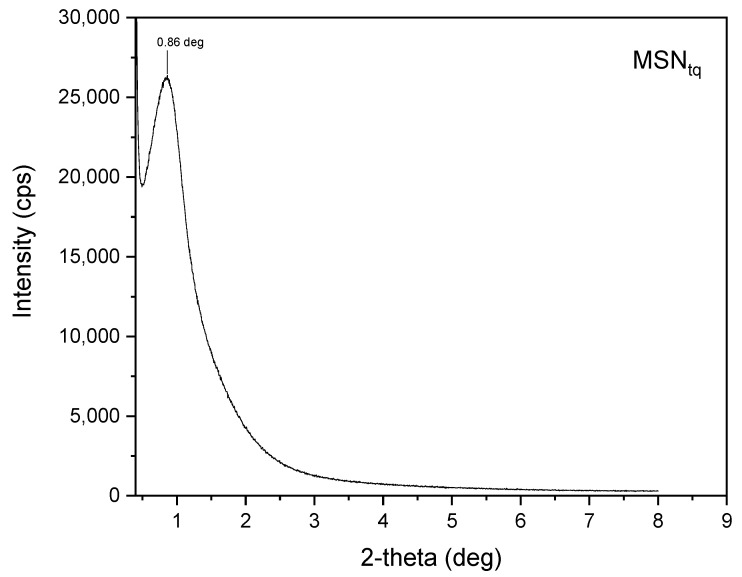
XRD pattern of synthesized MSU-x: MSN_AS_.

The small-angle XRD pattern (Figure 3) of the synthesized sample, MSN_AS_, demonstrated a single d100 diffraction peak at the range of 0–1°, typical of the family of the MSU-type materials [30].

The surfactant used as a templating agent was Triton-X100 (TX-100, polyethylene glycol p-(1,1,3,3,-tetramethylbutyl) phenylether), a nonionic surfactant that has a hydrophilic group of polyethylene oxide (on average it has 9.5 ethylene oxide units) and an aromatic lipophilic group. TX-100 was specifically used, and obviously generated an ordered mesoporous structure by acting as a template [31].

**Figure 4:**
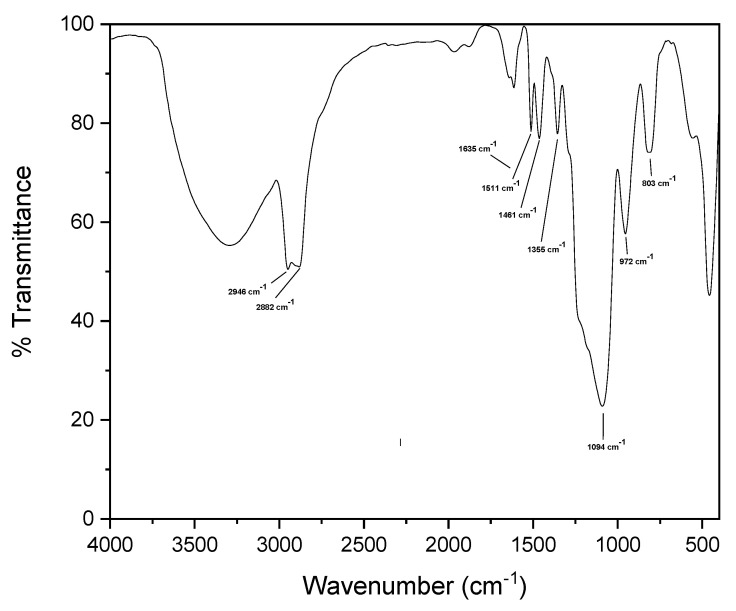
FTIR pattern of synthesized MSU-x: MSN_AS_.

The FTIR spectrum (Figure 4) revealed characteristic vibrational modes of mesoporous silica structures. At approximately 3400 cm^−1^, a broad absorption band was observed primarily due to the stretching of the different kind of OH groups and related interacting water, along with a sharp peak at 1635 cm^−1^. Vibrations at 2946 and 2882 cm^−1^ were attributed to the C-H stretching of methylene and methyl groups, respectively, of the TX-100 template’s. The bands at 1463 cm^−1^ and 1511 cm^−1^ corresponded to methyl asymmetric bending, overlapped with scissoring of the methylene groups, while the symmetrical bending vibration of methyl groups was observed at 1355 cm^−1^. The most intense band at 1094 cm^−1^, accompanied by a shoulder at 1210 cm^−1^, was attributed to the symmetric and antisymmetric stretching of the Si-O-Si structure, along with a less intense signal at 803 cm^−1^. Finally, silanol peaks arising from the vibrational bending modes were observed at 972 cm^−1^ [32,33].

**Figure 5:**
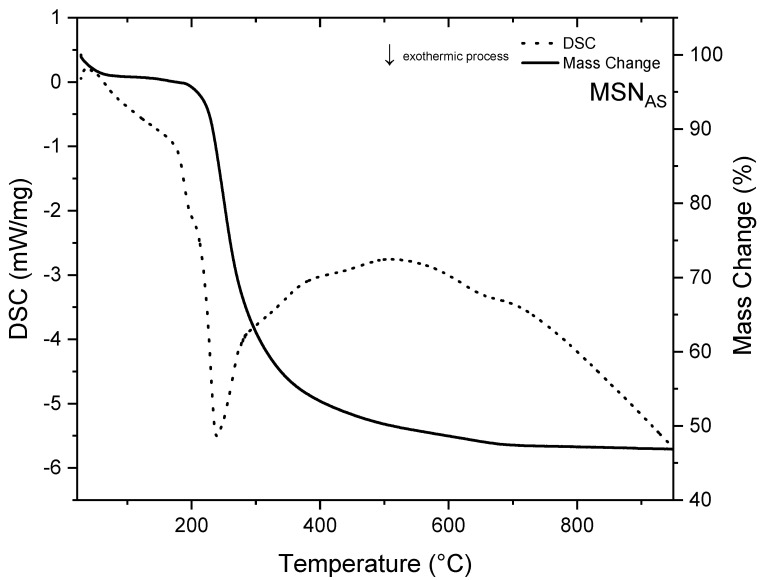
TGA-DSC pattern of synthesized MSU-x: MSN_AS_.

**Figure 6:**
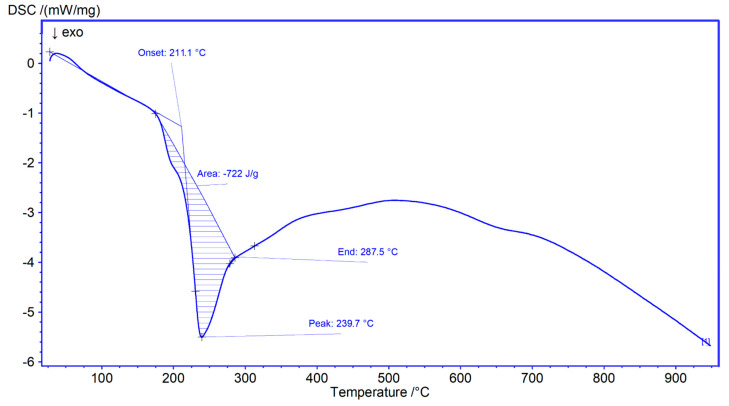
DSC: onset, end, peak, and enthalpy values.

TG/DSC analysis (Figure 5) of the MSN_AS_ sample showed a decrease of ~50% in mass, in the temperature range (200–950) °C, all attributable to TX-100 micelles, which decompose with an exothermic peak centered at 237.7 °C [34]. DSC values such as onset, peak, end, and enthalpy are depicted in Figure 6.

**Figure 7:**
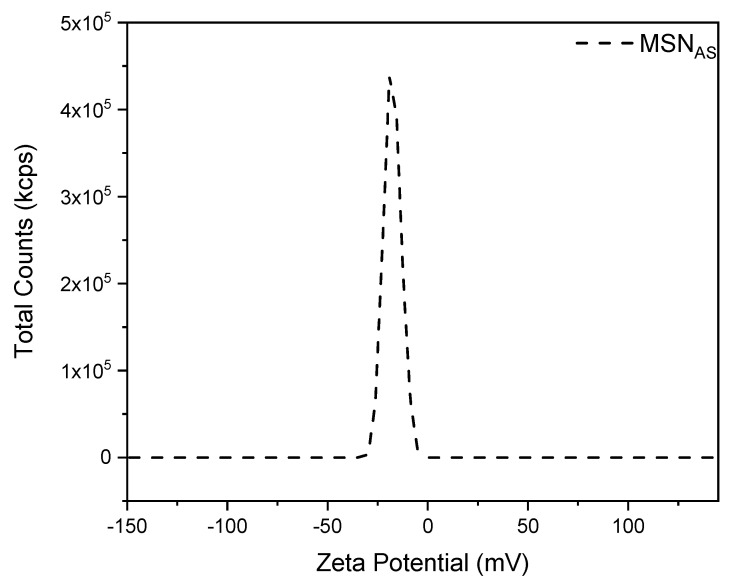
Zeta-potential pattern of synthesized MSU-x: MSN_AS_.

The MSN_AS_ samples exhibited zeta-potential values of approximately −18.00 ± 0.71 mV (Figure 7). Although absolute values below 30 mV may correlate with nanomaterials that exhibit a tendency to aggregate, it is important to note that zeta-potential values are not absolute indicators of suspension stability [35].

### 3.2. Functionalization of MSN_AS_ Sample and Characterization of Obtained Materials

**Scheme 2:**
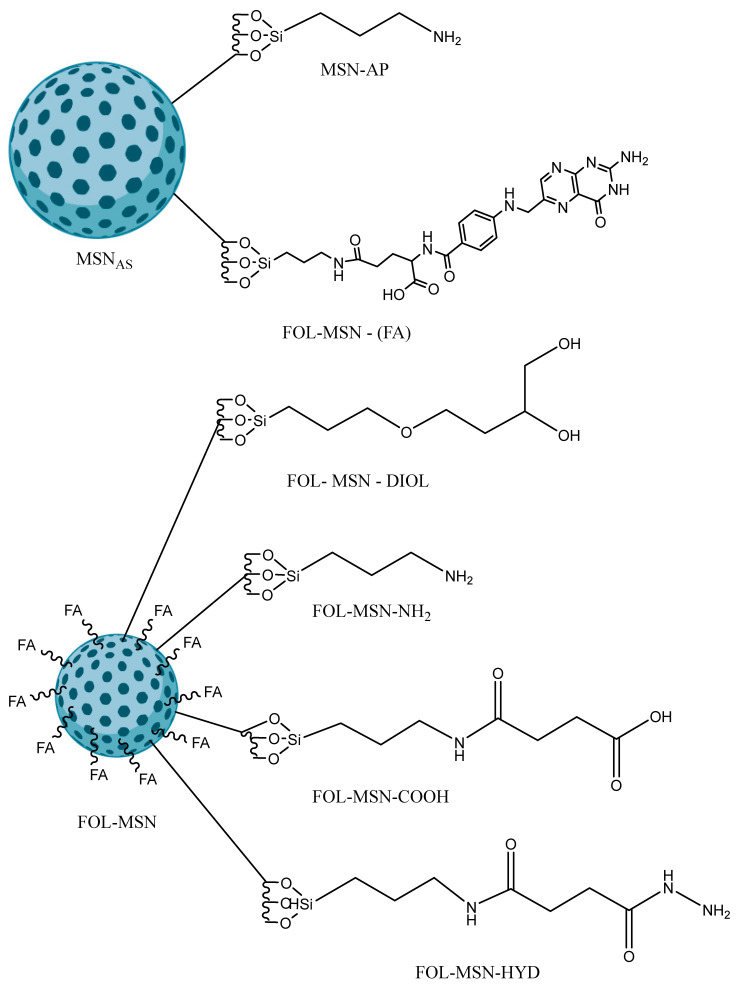
Graphical representation of grafting of MSN_AS_.

Several different functionalization protocols were followed to produce the different samples obtained, starting with the MSN_AS_ sample. (Scheme 2). Treatment of MSN_AS_ with APTES yielded MSN-AP, successively coupled with folic acid, to generate FOL-MSN. The implementation of a water extraction protocol facilitated the acquisition of FOL-MSN-EXT. The FOL-MSN-EXT sample underwent functionalization either with 3-glycidoxypropyltrimethoxysilane to obtain FOL-MSN-DIOL, either with APTES, resulting in FOL-MSN-NH_2_, followed by characterization. Starting with FOL-MSN-NH_2_, treatment with succinic anhydride led to the formation of FOL-MSN-COOH, followed by a reaction with hydrazine monohydrate to give FOL-MSN-HYD. Z-potential analysis, nitrogen adsorption porosimetry, and FTIR spectroscopy were employed to characterize all aforementioned samples.

### 3.3. Z-Potential Characterization

Determining the surface charge of nanostructured particles can be a challenging technological task. The most used technique involves determining the electric potential of a particle at a location away from the particle surface, somewhere in the diffuse layer. This position, correlated with particle movement in the liquid, is referred to as the slipping or shear plane. The potential measured at this plane is termed zeta potential, which is a critical parameter for colloidal or nanoparticle suspensions. The outcome of this determination is crucial in characterizing the suspension behavior of nanomaterials, as it correlates with the suspension stability as well as the size and morphology of the particles [36].

Moreover, the zeta potential (ζ) is fully determined by factors such as the surface nature, its charge typically influenced by pH, the concentration of electrolytes in the solution, as well as the characteristics of the electrolyte and the solvent. However, discrepancies can arise. These could be due to the high specific surface area and reactivity of colloidal systems, which make ζ highly sensitive even to minute amounts of impurities present in the solution. The zeta potential of suspended particles is measured after dilution to achieve high-resolution and accurate results [37].

High positive or negative values of the zeta potential indicate electrostatic interactions arising from surface charges. These interactions result in repulsive behaviors that prevent aggregation and flocculation. In general, zeta-potential values exceeding +30 or falling below −30 are associated with highly stable colloidal solutions. Additionally, the zeta-potential value is a crucial indicator for understanding the behavior of nanocomposite suspensions during the storage period. While the specific stability threshold may differ depending on the type of particles, assessing the stability of a sample holds significant importance in nanoparticle research. This is particularly crucial for various applications, such as minimizing aggregation for drug delivery and pharmaceutical purposes (requiring high zeta potential) or aiding in the removal of particles too small to be filtered out for water treatment applications (necessitating low zeta potential) [38,39,40]. Starting from modifications targeting the external surface of the nanoparticles (Figure 1), we first have the grafting of aminopropyl portions, followed by the linking of folic acid, and subsequently the extraction in ultrapure water of surfactant from the internal volume of the nanomaterial’s pores. Table 1 summarizes the Z-potential values of the analyzed samples.

**Figure 8:**
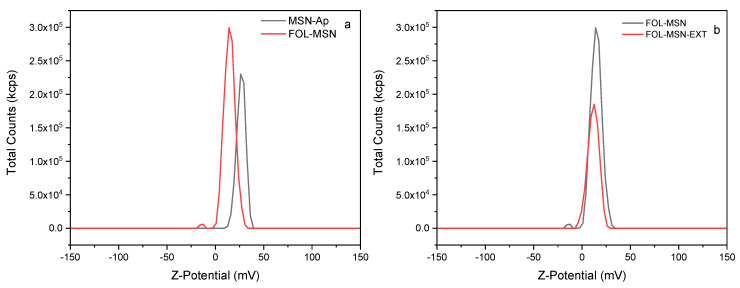
Z-potential graphs comparison of sample (**a**) MSN-Ap vs. FOL-MSN and (**b**) FOL-MSN vs. FOL-MSN-EXT.

As can be observed from the data summarized in Table 1, starting from negative zeta-potential (Z) values of approximately −18.00 mV for the precursor MSN_AS_ (Figure 7), primarily due to the presence of -OH groups on the nanomaterial surface [41], a positive Z value of about 27 mV was obtained in the sample subjected to surface grafting using 3-aminopropyltriethoxysilane (Figure 8a). This occurred because at pH 7, at which the zeta-potential measurements are recorded, the amino groups on the nanomaterial surface carry a positive charge, resulting in a positive surface charge [41,42]. Subsequent functionalization with folic acid exploits the chemistry of carbodiimides, in which folic acid is attached to the amino functionality by activating the carboxylic group of folate with N,N’-diisopropylcarbodiimide (DIC). The resulting amide is then bound to a suspension of MSN-AP, resulting in MSN-FOL (Figure 8a). Following this procedure, the surface charge on the nanoparticle is significantly reduced from +27 to +10 mV due to the reduction of the amino group previously present on the surface [43]. The subsequent removal of the template from the internal surface of the porous structure of the nanomaterial further releases silanol groups, but does not substantially affect the Z-potential value.

Surprisingly, contrary to what was hypothesized, the functionalization and subsequent hydrolysis of the FOL-MSN-DIOL sample yield a positive Z potential. This is likely due to the acid-base properties conferred by the presence of folic acid on the material, which play a crucial role in determining the Z-potential value, with its value being dependent on the acid-base characteristics of the surface [44]. Furthermore, it can be hypothesized that the FOL-MSN-DIOL sample, subjected to acid hydrolysis using a 0.001 N HCl solution starting from the FOL-MSN-GLY precursor, may have undergone protonation of the primary and secondary amine groups present in the folic portion. Even after the washing steps, at the pH at which the zeta-potential measurement was recorded, these groups remained protonated, generating a net positive charge that was reflected in the measured zeta-potential values.

**Figure 9:**
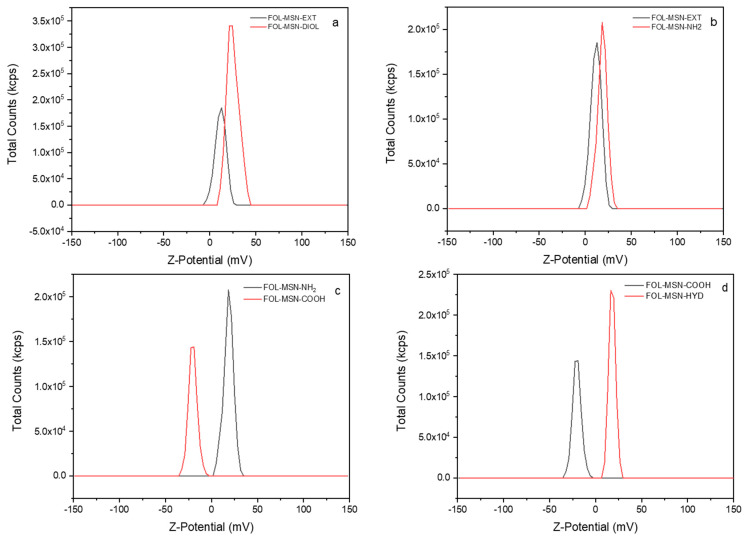
Z-potential graphs comparison of sample (**a**) FOL-MSN-EXT vs. FOL-MSN-DIOL; (**b**) FOL-MSN-EXT vs. FOL-MSN-NH_2_; (**c**) FOL-MSN-NH_2_ vs. FOL-MSN-COOH; (**d**) FOL-MSN-COOH vs. FOL-MSN-HYD.

Further functionalization with APTES in the FOL-MSN-NH_2_ sample (Figure 9b), as expected, returned the zeta-potential to positive values, whereas the subsequent modification of this precursor with succinic acid, owing to the presence of the -COOH group, led to more negative zeta-potential values (Figure 9c). The functionalizer imparts a negative charge to the surface [45,46]. As for the FOL-MSN-HYD sample (Figure 9d), it was synthesized by exploiting the formation of an amide between hydrazine and the free carboxylic group present. The corresponding zeta potential was influenced by the positive charge of the free amino groups present, which again increased the surface charge of the hybrid nanomaterial, bringing back positive values.

### 3.4. FTIR Characterization

**Figure 10:**
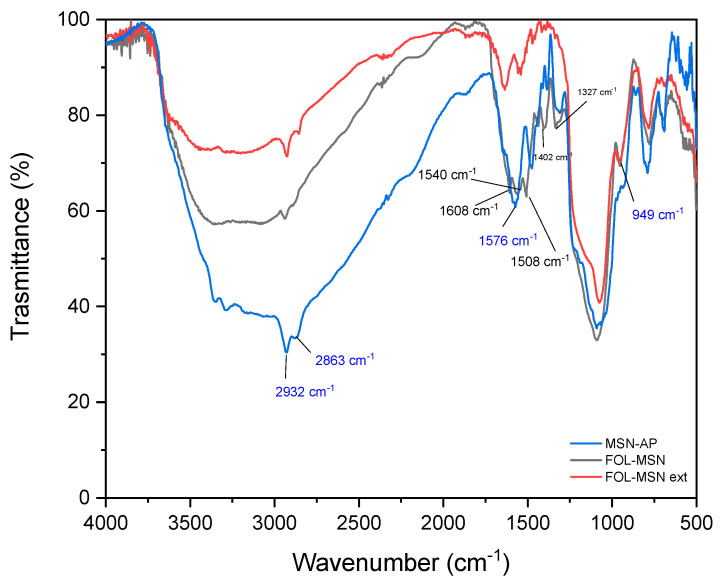
FTIR spectra overlap of MSN-AP, FOL-MSN, and FOL-MSN-EXT samples.

After aminopropyl-grafting of nanoparticles (MSN-AP), the spectrum (Figure 10) showed supplementary peaks. These included two absorption peaks within the 2932–2863 cm⁻¹ range, attributed to the stretching and bending vibrations, respectively, of the C-H bonds within the propyl group of organosiloxane. Additionally, a peak at 1576 cm⁻¹ was indicative of the N-H deformation modes characteristic of amine groups following the amination process [47]. Interestingly, peak at 949 cm^−1^, attributed to symmetric stretching of Si-OH, exhibited a lower intensity in aminopropyl-functionalized nanoparticles (AP-MSN), supporting the successful grafting processes.

The FTIR spectrum of the functionalized FOL-MSN exhibited a peak approximately at 1540 cm^−1^, attributed to the vibration of aromatic rings (ν C=C) within folic acid. The N–H stretching band (around 3400 cm^−1^) overlapped with the (ν O–H) band. Within the FOL-MSN spectrum, bands ranging from 1608 to 1508 cm^−1^ were assigned to the stretching of C=N, the bending of N–H, and the bending of O–H, which also overlap [48]. The functionalization performed on the internal surface of the porous structures of the nanomaterial occurred only following a process of extraction in ultrapure water of the surfactant used for the synthesis of MSN_AS_. The extraction allowed obtaining a pore volume free from other molecules, with reactive hydroxyl groups that can be used for silanization operations. The peaks at 1402 cm^−1^ and 1327 cm^−1^, assigned respectively to the asymmetric and symmetric bending of the methyl group related to TX-100, were no longer visible in the MSN-FOL-EXT sample.

**Figure 11:**
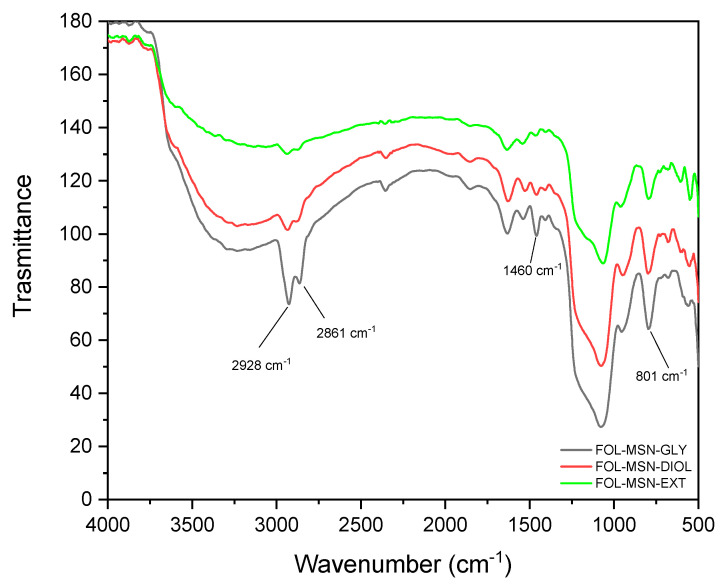
FTIR spectra overlap of MSN-FOL-EXT and MSN-FOL-GLY and MSN-FOL-DIOL samples.

The spectrum in Figure 11 represents the overlap of the MSN-FOL-EXT, MSN-FOL-GLY, and its hydroxylated derivative MSN-FOL-DIOL samples. It can be observed that the differences are summarized by a higher presence of peaks at around 2928–2861 cm^−1^, which are assignable to the symmetric and antisymmetric vibration of CH and CH_2_ of the epoxide, more pronounced in the non-hydrolyzed sample. Similarly, the peak at around 801 cm^−1^, which is typical of the epoxide ring vibration, which also overlaps with the peak assigned to the stretching of the Si-O-Si bond, is more pronounced in the non-hydrolyzed sample [49].

Furthermore, there is a more intense peak at 1460 cm^−1^ in the FOL-MSN-GLY and FOL-MSN-DIOL samples compared to the FOL-MSN-EXT sample. This could likely be assigned to the bending of the methylene groups in the alkyl chain of the 3-glycidoxypropyltrimethoxysilane substituent.

**Figure 12:**
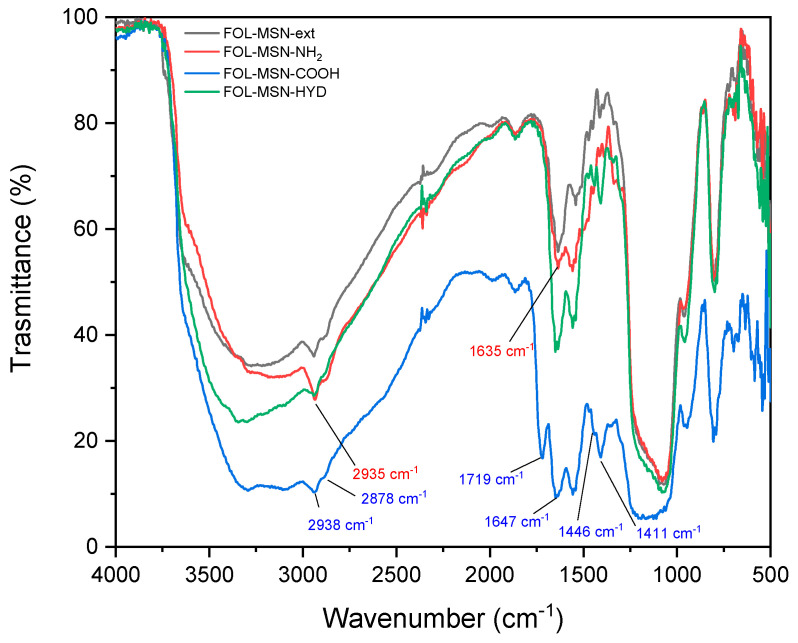
FTIR spectra overlap of FOL-MSN-EXT, FOL-MSN-NH_2_, FOL-MSN-COOH, and FOL-MSN-HYD samples.

In Figure 12, the overlap between the FOL-MSN-EXT sample and the sample further functionalized with the addition of 3-aminopropyltriethoxysilane FOL-MSN-NH_2_ is evident. Since the grafting reaction occurs on a sample whose internal pores have been freed from the previously present surfactant through successive extractions in ultrapure water, we can reasonably assume that APTES primarily bonded with the free -OH groups present within the pores of the FOL-MSN-EXT nanomaterial. The FT-IR spectrum of FOL-MSN-COOH exhibited a broad transmittance band at approximately 3400–3200 cm^−1^ due to -OH and -NH stretching, and weak bands at 2938 and 2878 cm^−1^ due to -CH stretching frequencies. The stretching bands of amidic carbonyl and carboxylic carbonyl (C=O) occurred at approximately 1650 cm^−1^ and 1700 cm^−1^, respectively. The -NH bending vibration was observed at 1446 cm^−1^, and the -CH bending vibrations were observed at around 1411 cm^−1^. The presence of these transmittance bands indicates the presence of amidic succinic acid moiety on the surface of FOL-MSN-COOH [50]. For the FOL-MSN-HYD sample, it can be observed that this exhibit showed almost overlapping bands and peaks with FOL-MSN-COOH, as both samples carry very similar chemical functionalities. The differences that can be observed include, for example, the presence of a much more intense band in the range of 3400–3200 cm^−1^, and were mainly due to the stretching of the free -OH groups present in the carboxylic functionality of FOL-MSN-COOH and less intense in FOL-MSN-HYD. Peaks at around 1600–1650 cm^−1^, typical of carbonyl stretching of amides, were present in both spectra as functionalities in both samples. Anyway, the most noticeable difference in this spectrum cannot be shown because the peak at around 1110 cm^−1^, which is typical of the stretching of the N-N bond of the free hydrazine functionality, was hidden by another absorption band [51].

However, the clear presence of the hydrazine functionality was confirmed by the zeta-potential results, which returned to very positive values following the grafting reaction.

### 3.5. Nitrogen Adsorption Characterization

**Figure 13:**
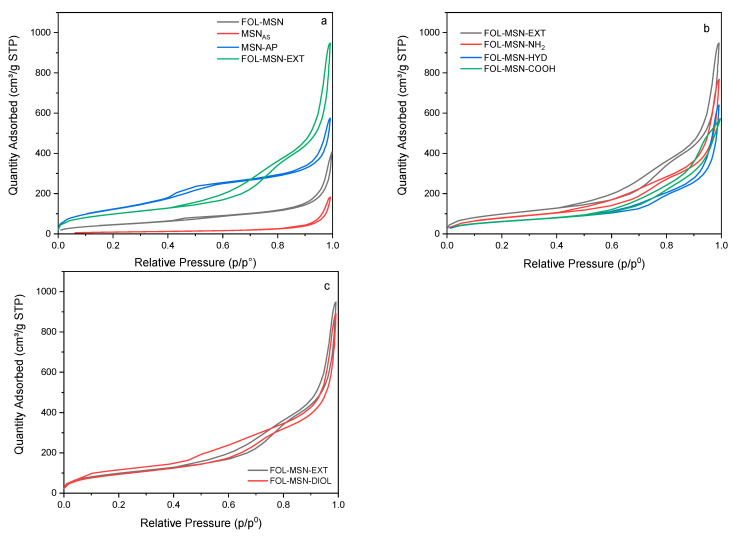
Adsorption isotherm overlap of (**a**) MSN_AS_, MSN-AP, FOL-MSN and FOL-MSN-EXT samples; (**b**) FOL-MSN-NH_2_, FOL-MSN-EXT, FOL-MSN-COOH, and FOL-MSN-HYD samples; (**c**) FOL-MSN-EXT vs. FOL-MSN-DIOL samples.

In Figure 13a, the overlap of the nitrogen adsorption-desorption graphs of the MSN_AS_, MSN-AP, FOL-MSN, and FOL-MSN-EXT samples reveals that the first three samples obviously did not exhibit any porous structure, as they still contained the template used in the synthesis process within their pores. As expected, MSN-AP was slightly less occluded than the FOL-MSN sample, both because during the synthesis protocol of MSN-AP, the precursor was suspended in a solution of APTES-ethanol which partially extracted the template. Regarding the FOL-MSN-EXT sample, the adsorption curve presented a type IV isotherm, typical of mesoporous materials, and as expected, compared to the other samples, the surface area increased significantly, due to the extraction of the template from the mesoporous structure. However, these data were not very high in absolute value, and one must take into account the contribution of the organic fraction to the mass of the hybrid mesoporous material and the low activation temperature of the sample to preserve the folate functionality; this probably led to the retention of water within the porous structure [52].

BET surface, pore volume, and pore width values are summarized in Table 2.

**Scheme 3:**
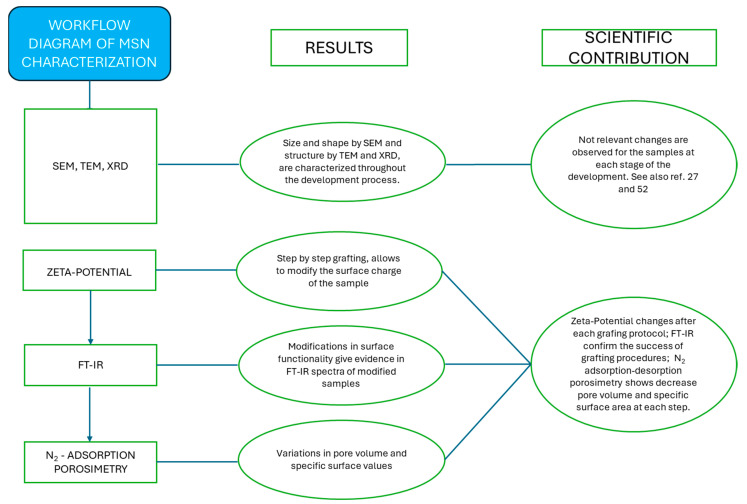
Flow chart of results and scientific contribution.

In Scheme 3, the results acquired in the presented multistep development of hybrid nanostructured materials are connected with their scientific contribution. It can be ob-served that the higher significance derived from zeta-potential, FT-IR spectroscopy, and nitrogen adsorption-desorption analyses. These analyses, in fact, related to a relevant scientific contribution to the characterization of the obtained materials. The electronic mi-croscopies (SEM and TEM), powder X-ray diffraction, and 29Si-NMR spectroscopy, on the other side, were shown to be not significantly affected by multistep modification procedures, as we showed in detail in our previous works [27,52]. The results revealed significant variations in the surface characteristics of MSNPs following each post-synthesis operation. Zeta- potential measurements indicated changes in surface charge distribution, suggesting alterations in the material’s colloidal stability and potential interactions with surrounding environments.

Both single-step and multi-step grafting procedures induced notable modifications in surface functionality, as evidenced by shifts in infrared absorption bands associated with functional groups. These findings underscore the versatility of grafting techniques in tailoring surface chemistry, potentially enhancing the material’s applicability in diverse fields such as drug delivery and environmental remediation.

Furthermore, surfactant extraction demonstrated a significant influence on surface porosity, as expected, and as indicated by changes in nitrogen adsorption isotherms. The observed variations in pore size distribution highlight the importance of surfactant removal in optimizing the material’s pore accessibility, a crucial factor in controlling drug release kinetics and pollutant adsorption capacity. 

## 4. Conclusions

This article specifically investigated the surface properties of mesoporous silica nanoparticles, focusing on functionalization processes and their implications. Utilizing various characterization techniques, the study elucidated how surface modifications influence the zeta potential and functional group composition, crucial in understanding colloidal behavior and applications.

The research delineated a systematic approach to surface functionalization, starting from precursor materials and progressing through successive modifications. The sequence of functionalization steps produced the expected surface functionalities whose presence was confirmed by FT-IR spectroscopy; on the other hand, the rapid changing of the zeta-potential values gave an indirect confirmation that these versatile functionalization procedures were successful. Overall, we consider these potentialities as valid instruments to define at the nanoscale the surface of a nanostructured material both for precise application in the field of environmental remediation and in nanomedicine applications, where an increasing awareness on nano−bio interfaces can enhance in vivo performance and facilitate clinical translation of nanomaterials/nanomedicines [53].

This paper provides insights on the effects of post-synthesis surface treatments on the properties of MSNPs. By connecting the observed changes with established theories of surface modification and nanoparticle behavior, this study advances the understanding of nanomaterial design principles and lays the groundwork for further exploration in related fields.

## Figures and Tables

**Table 1:** Z-potential values of the analyzed samples.

Sample	Z-Potential Value (mV)	Std. Dev. (mV)
MSN_AS_	−18.0	±0.71
MSN-AP	+27.7	±5.19
FOL-MSN	+10.5	±5.74
FOL-MSN-EXT	+9.91	±4.81
FOL-MSN-DIOL	+25.50	±6.18
FOL-MSN-NH_2_	+18.40	±3.80
FOL-MSN-COOH	−19.10	±3.40
FOL-MSN-HYD	+16.90	±3.59

**Table 2:** N_2_ adsorption data.

Sample	BET Surface Area (m^2^∙g^−1^)	Pore Volume (cm^3^/g) At P/P^0^ = 0.96	Average Pore Width Desorption Branch (BJH) (nm)
MSN-AP	250.45	0.615	11.55
FOL-MSN	207.45	0.361	11.04
FOL-MSN-EXT	423.38	1.512	16.67
FOL-MSN-DIOL	349.02	0.820	12.33
FOL-MSN-NH_2_	295.09	1.187	15.83
FOL-MSN-COOH	224.47	0.675	12.40
FOL-MSN-HYD	226.52	0.572	16.67

## Data Availability

The original contributions presented in the study are included in the article, further inquiries can be directed to the corresponding author.
